# Influence of late gadolinium enhancement on left ventricular morphology and function in patients with sarcoidosis

**DOI:** 10.1186/1532-429X-11-S1-O64

**Published:** 2009-01-28

**Authors:** Amit R Patel, Nadera J Sweiss, Sonal Chandra, Lissa Sugeng, Kirk T Spencer, Jeanne M DeCara, Martin C Burke, Timothy B Niewold, Douglas K Hogarth, Stephen L Archer, John F Beshai

**Affiliations:** University of Chicago, Chicago, IL USA

**Keywords:** Sarcoidosis, Cardiac Magnetic Resonance, Late Gadolinium Enhancement, Left Ventricular Mass, Cardiac Involvement

## Background

Cardiac involvement in sarcoidosis patients is associated with an increased risk of sudden death. However, conduction block and cardiomyopathy are late and therefore potentially insensitive markers of cardiac sarcoidosis. Prior publications have suggested that the detection of late gadolinium enhancement (LGE) by cardiac magnetic resonance (CMR) may be a more sensitive parameter for diagnosing cardiac sarcoidosis. Using CMR, in patients with known sarcoidosis, we sought 1) to further define the incidence of cardiac involvement and 2) to determine the correlation between LGE and structural cardiac pathology.

## Methods

We retrospectively evaluated 55 consecutive patients with known sarcoidosis who were referred for CMR. Imaging was performed on a 1.5 Tesla MRI scanner with a flexible surface coil. Retrospectively gated cines of the left ventricular (LV) 2-, 3-, and 4-chambers, and a short-axis stack were obtained using steady state free precession imaging (TR 2.9 ms, TE 1.5 ms, flip angle 60°, temporal resolution 25–40 ms). LGE images of the same views were obtained 10–20 minutes after infusion of Gd-DTPA (0.15–0.2 mmol/kg) using a T1-weighted inversion recovery GRE pulse sequence (TI based on optimal myocardial nulling, TR 3.9 ms, TE 1.7 ms, flip angle = 15–30°). The cines of the short-axis stack were used to determine LV and right ventricular end-diastolic volume (LVEDV and RVEDV), end-systolic volume (LVESV and RVESV), ejection fraction (LVEF and RVEF), and LV mass. The presence or absence of LGE was determined for each of the 17 segments of the American Heart Association left ventricular model. Continuous variables were reported as mean ± standard deviation, groups (based on the presence or absence of LGE) were compared using a t-test, and linear regression analysis was used to investigate the correlation between LGE and LV size and function. A p value < 0.05 was considered statistically significant.

## Results

**Figure 1:**
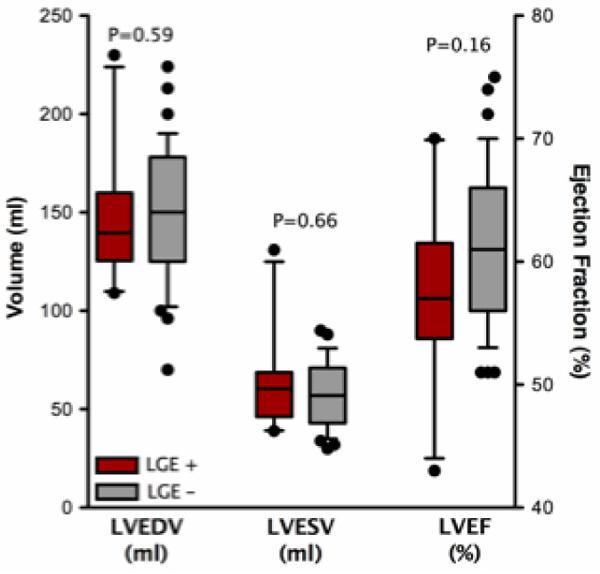
Figure 1

**Figure 2:**
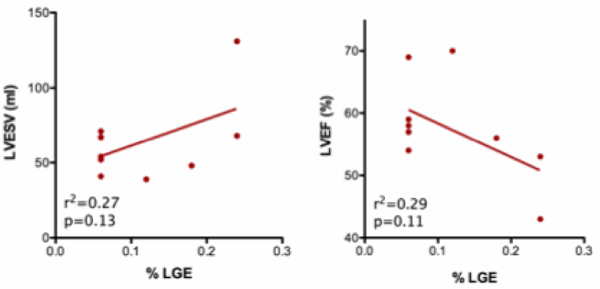
Figure 2

The patients were 48 ± 13 years old and the majority were women (73%). Twenty-two percent of the patients had LGE involving on average 14 ± 12% of the LV. In patients with or without LGE, there was no difference in LVEDV (147 ± 35 ml vs. 150 ± 35 ml, p = 0.59), LVESV (64 ± 16 ml vs. 57 ± 16 ml, p = 0.66), LVEF (58 ± 8% vs. 62 ± 7%, p = 0.16), LV mass (116 ± 46 g vs. 99 ± 24 g, p = 0.47), RVEDV (172 ± 35 ml vs. 171 ± 46 ml, p = 0.91), RVESV (85 ± 26 ml vs. 82 ± 27 ml, p = 0.78) and RVEF (52 ± 6% vs. 53 ± 7%, p = 0.70). See figure 1. Moreover, based on linear regression analysis, in the patients with LGE, the extent of enhancement did not influence LVEDV (r^2^ = 0.14, p = 0.29) and LV mass (r^2^ = 0.03, p = 0.65). However, there was a trend that the extent of LGE correlated with both LVESV (r^2^ = 0.27, p = 0.13) and LVEF (r^2^ = 0.29, p = 0.11) (See figure 2). All of the above relationships persisted even after accounting for body surface area.

## Conclusion

In patients with known sarcoidosis, nearly a quarter had evidence of cardiac involvement, as defined by the presence of LGE, despite the lack of any difference in traditional measures of cardiac structure and function. These preliminary findings suggest that volumetric and functional assessments alone are inadequate for the detection of cardiac sarcoidosis and that tissue characterization using LGE is essential. Further studies are needed to determine the long-term prognostic value of LGE and to further clarify the relationship between the extent of LGE and left ventricular morphology and physiology in patients with cardiac sarcoidosis.

